# Genome-Wide Identification and Functional Analysis of the *TIFY* Family Genes in Response to Abiotic Stresses and Hormone Treatments in Tartary Buckwheat (*Fagopyrum tataricum*)

**DOI:** 10.3390/ijms241310916

**Published:** 2023-06-30

**Authors:** Zhixing Zhao, Guanghua Meng, Imran Zamin, Tao Wei, Dongdi Ma, Lizhe An, Xiule Yue

**Affiliations:** 1 Ministry of Education Key Laboratory of Cell Activities and Stress Adaptations, School of Life Sciences, Lanzhou University, Lanzhou 730000, China; zhaozhx2014@lzu.edu.cn (Z.Z.); imran.zamin@gmail.com (I.Z.); weit2020@lzu.edu.cn (T.W.); 220220933570@lzu.edu.cn (D.M.); 2 School of Life Sciences and State Key Laboratory of Agrobiotechnology, The Chinese University of Hong Kong, Shatin, New Territories, Hong Kong 999077, China; ghmeng1302@gmail.com; 3 The College of Forestry, Beijing Forestry University, Beijing 100000, China

**Keywords:** Tartary buckwheat, *TIFY* gene family, abiotic stress, JAZ protein, heat tolerance

## Abstract

*TIFY* is a plant-specific gene family with four subfamilies: *ZML*, *TIFY*, *PPD*, and *JAZ*. Recently, this family was found to have regulatory functions in hormone stimulation, environmental response, and development. However, little is known about the roles of the *TIFY* family in Tartary buckwheat (*Fagopyrum tataricum*), a significant crop for both food and medicine. In this study, 18 *TIFY* family genes (*FtTIFYs*) in Tartary buckwheat were identified. The characteristics, motif compositions, and evolutionary relationships of the TIFY proteins, as well as the gene structures, *cis*-acting elements, and synteny of the *TIFY* genes, are discussed in detail. Moreover, we found that most *FtTIFYs* responded to various abiotic stresses (cold, heat, salt, or drought) and hormone treatments (ABA, MeJA, or SA). Through yeast two-hybrid assays, we revealed that two FtTIFYs, FtTIFY1 and FtJAZ7, interacted with FtABI5, a homolog protein of AtABI5 involved in ABA-mediated germination and stress responses, implying crosstalk between ABA and JA signaling in Tartary buckwheat. Furthermore, the overexpression of *FtJAZ10* and *FtJAZ12* enhanced the heat stress tolerance of tobacco. Consequently, our study suggests that the *FtTIFY* family plays important roles in responses to abiotic stress and provides two candidate genes (*FtJAZ10* and *FtJAZ12*) for the cultivation of stress-resistant crops.

## 1. Introduction

*TIFY* is a plant-specific gene family that exhibits regulatory functions in plant development, hormone stimulation, and environmental response. This family derives its name from a conserved core motif (TIF[F/Y]XG) situated in a 36-amino-acid domain called TIFY and can be classified into four subfamilies according to their distinct domain constitutions: TIFY, JAZ (JASMONATE ZIM-domain), ZML (ZIM and ZIM-like), and PPD (PEAPOD) [1,2]. Notably, the TIFY subfamily lacks any additional domains, whereas the other three subfamilies possess multiple domains alongside the TIFY domain. The JAZ subfamily comprises a TIFY domain and a JAS domain (SLX2FX2KRX2RX5PY), which exhibits sequence similarities with the N-terminal sequence of the CCT domain [3,4]. Within the ZML subfamily, a TIFY domain is accompanied by a CCT domain (CON-STANS, CO-like, TOC1) and a GATA zinc finger domain [1,3,4,5]. Lastly, the PPD subfamily proteins encompass a TIFY domain, a PPD domain, and a truncated Jas motif lacking conserved P and Y residues [4].

Since their identification in *Arabidopsis thaliana*, a rising number of studies have reported on the functions of *TIFYs* in plant growth, phytohormone response, and stress tolerance [6]. The JAZ subfamily proteins have emerged as crucial components of the jasmonate (JA) signaling pathway, acting as transcriptional inhibitors. When plants encounter phytohormones or stress stimuli, the F-box protein coronatine insensitive 1 (SCF^COI1^) recognizes JA-coupled isoleucine (JA-ile), triggering the degradation of JAZ proteins and subsequently activating JA-responsive genes [7]. However, the up-regulation of JAZ genes was observed upon stimulation with JA or abiotic stresses, indicating the presence of a feedback regulatory mechanism that replenishes the reservoir of JAZ proteins while dampening the response to JA. This intricate interplay contributes to the development of heightened stress tolerance [8,9,10,11].

Indeed, the overexpression of *GsJAZ2* has been shown to improve salt and alkali resistance in soybeans [12]. The salt-induced overexpression of *OsJAZ8* demonstrated superior tolerance to salt stress compared to the wild type during early rice development [13]. Additionally, *OsJAZ1* and *GaJAZ5* were implicated in the drought stress tolerance of rice and cotton [14,15]. Except for the JAZ subfamily proteins, the members of the *TIFY* subfamily also participate in stress tolerance. The overexpression of *GsTIFY10* enhances bicarbonate stress tolerance in wild soybeans [16]. OsTIFY11a, a transcriptional regulator in rice, forms a regulatory complex with OsbHLH and OsNINJA to control salt tolerance [17,18].

Instead, the ZIM and PPD subfamily proteins are most likely involved in plant growth. The overexpression of *ZIM* boosted petiole and hypocotyl cell elongation during plant photomorphogenesis, regardless of gibberellin and brassinosteroids [19], whereas *AtPPD1/2* was reported to regulate leaf development by arresting dispersed meristematic cell proliferation, and its loss-of-function mutant enhanced leaf size and dome shape [20,21,22].

Tartary buckwheat (*Fagopyrum tartaricum*), a member of the Polygonaceae family, holds considerable significance as a nutritive and medicinal plant that is indigenous to China. It is replete with an assortment of carbohydrates and flavonoids, particularly rutin, exhibiting significant antioxidant, anti-inflammatory, anti-carcinogenic, and cardiovascular-preventive properties [23]. Given its natural occurrence in Southwest China and its prevalence along the fringes of the Himalayas, Tartary buckwheat has attracted the attention of numerous scientific researchers, who have documented its robust adaptability to abiotic stressors [24,25,26]. We hypothesized that the unexplored TIFY protein family, which is exclusive to plants, plays a pivotal role in the growth, hormonal responses, and, in particular, the stress reactions of Tartary buckwheat.

In this investigation, a comprehensive examination was undertaken to identify and characterize 18 *TIFY* family genes (*FtTIFYs*) within the assembled genome of Tartary buckwheat [24]. The aim was to shed new light on their evolutionary relationships and potential functions by assessing their characteristics, motif compositions, evolutionary connections, gene structures, synteny patterns, and *cis*-element properties. Meanwhile, the expression profiles of *FtTIFYs* in response to various abiotic stresses and hormone treatments were determined through quantitative RT-PCR analysis. Next, we elucidated the potential association between the TIFY-mediated JA signaling pathway and the resistance of Tartary buckwheat to abiotic stresses by employing in vivo protein–protein interaction studies. To further confirm the crucial role of FtTIFYs in response to abiotic stress, we demonstrated that the transient overexpression of two JAZ proteins in tobacco leaves effectively enhances stress tolerance under heat conditions. In summary, this study represents an initial investigation of the response of *TIFY* genes to environmental stresses and exogenous hormone treatment, thereby facilitating functional characterization research on *TIFY* genes in Tartary buckwheat.

## 2. Results

### 2.1. Identification of TIFY Family Genes in Tartary Buckwheat

Eighteen putative *TIFY* genes were detected in the genome of Tatary buckwheat using BLAST and HMMER searches. The conserved domains in their coding protein sequences were further examined using Pfam. Based on the motif and domain composition, the eighteen TIFYs were further categorized into four subfamilies. One protein with only one TIFY domain belonged to the TIFY subfamily and was designated FtTIFY1. The remaining seventeen proteins possessed the Jas motif in addition to the TIFY domain. Of these Jas-motif-containing proteins, twelve that only have a TIFY domain and a Jas motif belonged to the JAZ subfamily and were designated as FtJAZ1 to FtJAZ12. Two of the remaining proteins without the P and Y residues in the Jas motif, which rendered them identical to the PDD subfamily, were designated as FtPPD1 and FtPPD2. The three remaining proteins containing the TIFY domain, GATA zinc finger, Jas motif, and CCT motif belonged to the ZML subfamily and were designated as FtZML1 to FtZML3. Among the TIFY proteins of Tatary buckwheat, FtJAZ10 is the smallest protein (114 aa), and the largest protein is FtJAZ11 (554 aa) (Table 1). The molecular weight of the proteins ranges from 12.91 kDa to 57.47 kDa; the pI ranges from 6.28 (FtZML3) to 11.00 (FtJAZ3).

### 2.2. Phylogenetic Analysis and Classification of the FtTIFYs

**Figure 1:**
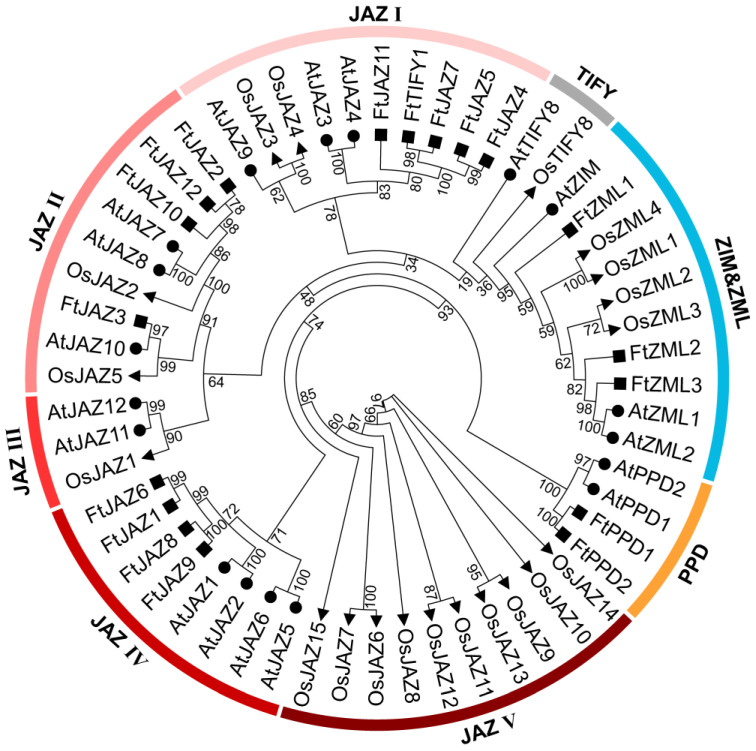
Phylogenetic tree of TIFY proteins from Tartary buckwheat (18 TIFYs), *Arabidopsis thaliana* (18 TIFYs), and rice (20 TIFYs). The maximum likelihood (ML) phylogenetic tree was built using IQtree based on 56 TIFY protein sequences (1000 bootstrap replicates).

To explore the evolutionary relationship of JAZ, TIFY, ZML, and PPD in Tartary buckwheat, rice, and *Arabidopsis thaliana*, a phylogenetic tree containing 18 AtTIFY proteins, 20 OsTIFY proteins, and 18 FtTIFY proteins was created. Based on the results of the phylogenetic analysis and the classification of TIFYs in other species [10,27,28], the TIFY proteins were classified into eight groups, including JAZ I-V, PPD, TIFY, and ZML (Figure 1). The investigation produced several intriguing findings. For instance, both Tartary buckwheat and *Arabidopsis thaliana* contributed two PPD proteins to the PPD subfamily, three ZML/ZIM proteins to the ZML subfamily, and four JAZ proteins to the JAZ IV group, indicating that neither clade has undergone major expansion or contraction since the divergence of monocots (rice) and eudicots (Tartary buckwheat and *Arabidopsis thaliana*). Among the JAZ subfamily, JAZ I and II comprised JAZ proteins from *Arabidopsis thaliana*, rice, and Tartary buckwheat; JAZ Group III had JAZ proteins from rice and *Arabidopsis thaliana*; and JAZ Group V contained JAZ proteins from rice alone. These results indicated that the FtTIFY proteins were more closely linked to *Arabidopsis thaliana* TIFY proteins than to rice TIFY proteins. This finding is consistent with the fact that both Tartary buckwheat and *Arabidopsis thaliana* are eudicots and have more recently diverged from a common ancestor. Interestingly, the FtTIFY1 protein was clustered in the JAZ I group, suggesting that it probably was a JAZ protein that lost the Jas domain during evolution.

### 2.3. Chromosomal Distribution and Synteny of the FtTIFY Genes

**Figure 2:**
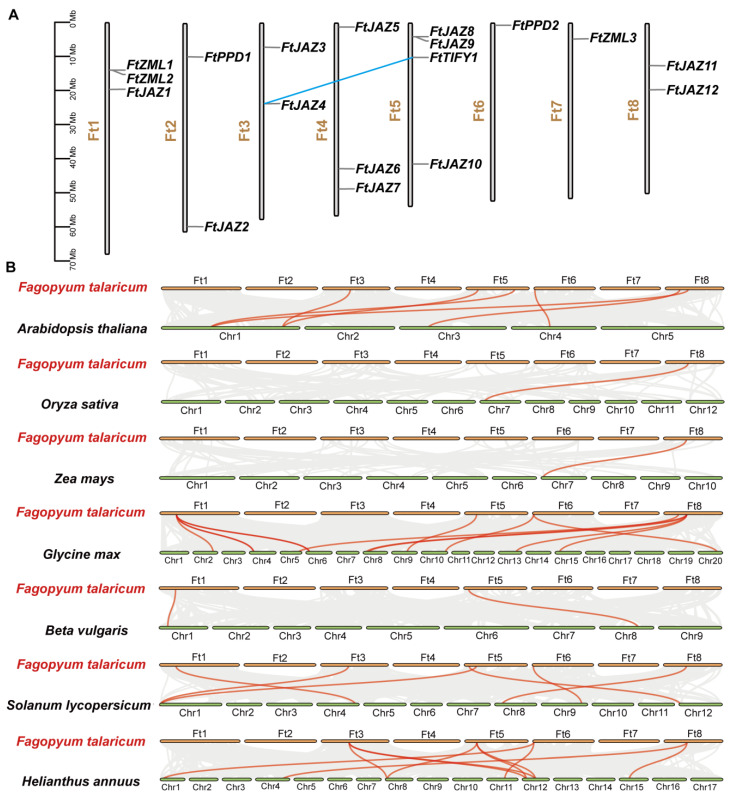
Chromosomal distribution and synteny analysis of *FtTIFY* genes. (**A**) Locations and segmental duplication of *TIFY* genes in Tartary buckwheat genome. One pair of the segmental duplicated gene is shown by the blue line. (**B**) Synteny analysis of *TIFY* genes between Tartary buckwheat and seven representative plant species. Grey lines in the background indicate collinear blocks in the genomes of Tartary buckwheat and other plants. Red lines represent the syntenic *TIFY* gene pairs.

The chromosome locations and synteny of the *FtTIFY* genes were analyzed. Eight chromosomes of Tartary buckwheat contained an unequal distribution of 18 *FtTIFY* genes. Chromosome 5 contained the most *FtTIFY* genes (four), while chromosomes 6 and 7 contained the fewest (only one) (Figure 2A). The synteny analysis showed that only one pair of genes, *FtTIFY1* and *FtJAZ4*, displayed the segmental duplication event in the Tartary buckwheat genome, and the Ka/Ks ratio of *FtTIFY1/FtJAZ4* was <1 (Table S3), suggesting potential negative selection [29]. We performed a further synteny analysis of Tartary buckwheat and the other seven species (*Arabidopsis thaliana*, rice, maize, soybean, beet, tomato, and sunflower) (Figure 2B). The results revealed that the *FtTIFY* genes had homologous gene pairs in all seven species. The numbers of homologous gene pairs between Tartary buckwheat and the seven species identified above were 6, 1, 1, 13, 2, 6, and 10, respectively (Figure 2B and Table S4). Notably, some *FtTIFY* genes were linked to three or more genes, particularly between Tartary buckwheat and soybean *TIFYs*. For instance, *FtJAZ12* has four pairs of homologous genes, while *FtZML1* has three pairs, indicating that these genes may have played crucial roles in evolution. However, there is only one homologous gene pair between Tartary buckwheat and rice/maize, implying that *TIFY* is much more conserved in dicots.

### 2.4. Conserved Motifs, Gene Structure, and Cis-Element Analysis of the FtTIFYs

**Figure 3:**
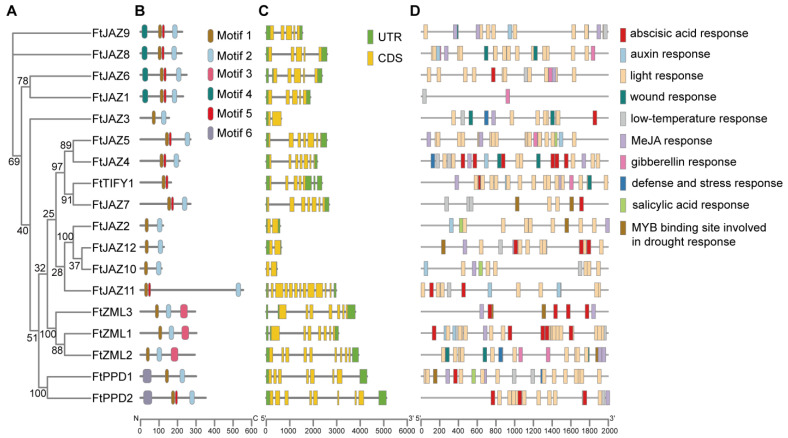
Sequence analysis of FtTIFYs. (**A**) Phylogenetic analysis of FtTIFY proteins with maximum likelihood method using IQtree with 1000 bootstrap replicates. (**B**) Distribution of conserved motifs among the FtTIFY proteins. Six motifs are marked with different colored boxes. (**C**) Structure of exons/introns in *FtTIFY* genes. Untranslated regions (UTRs), exons, and introns are shown by the green boxes, yellow boxes, and grey lines, respectively. (**D**) The predicted *cis*-elements in *FtTIFY* promoter regions. Ten elements are represented by blocks of different colors.

Further phylogenetic analysis was conducted exclusively with FtTIFY sequences. The results showed a similar topology to that of the phylogenetic tree built from the TIFYs of the three above-mentioned plants (Figure 1 and Figure 3A). Motif analysis of the FtTIFY proteins using the MEME server indicated that proteins in the same clade shared significant similarities in the protein length and distribution patterns of the conserved motifs. Six motifs were discovered in the FtTIFY proteins. In addition to the TIFY domain, consisting of motif 1 or motifs 1 and 5, seventeen FtTIFY proteins (with the exception of FtTIFY1) contained motif 2 (the Jas motif). FtPPD1 and FtPPD2 had motif 6 (the PPD domain), but FtZML1, FtZML2, and FtZML3 had motif 3 (the GATA zinc finger domain). In addition, FtJAZ1, FtJAZ6, FtJAZ8, and FtJAZ9 contained a motif 4 of unknown function (Figure 3B and Figure S1).

The gene structures of the 18 *FtTIFYs* were then investigated to examine their characteristics. The results revealed that the phylogenetically related *FtTIFY* genes possessed similar exon/intron structures. For example, six introns were found in *FtTIFY1*, *FtJAZ4*, *FtJAZ5*, and *FtJAZ7*; four introns were found in *FtJAZ1*, *FtJAZ8*, and *FtJAZ9*; and only one intron was found in *FtJAZ2*, *FtJAZ3*, *FtJAZ10*, and *FtJAZ12* (Figure 3C), indicating that the three sets of *FtTIFYs* might be the result of gene duplications. 

Furthermore, we found evidence of exon/intron gain in several *FtTIFYs.* For instance, *FtZML2* contains two more exons than all the other *FtZML* genes, and *FtJAZ11* has at least five more exons than the rest of the *FtJAZ* genes (Figure 3C). This may be a result of transposable element insertion during the evolution [30]. 

To identify the putative *cis*-elements that regulate the transcription of *FtTIFY* genes, a 2 kb promoter region of each *FtTIFY* gene was analyzed with the PlantCARE database [31]. The results showed that most of the promoters comprised diverse putative phytohormones or stress-responsive elements (Figure 3D). For example, abscisic acid-responsive *cis*-elements were present in 16 *FtTIFY* genes. The *cis*-elements related to MeJA, auxin, gibberellin, and SA responses were found in the promoters of 15, 9, 7, and 4 *FtTIFY* genes. In addition, the *cis*-elements involved in low temperature, drought, immunity, and stress responses were identified in the promoters of the 12, 6, and 5 *FtTIFY* genes. These findings suggested that *FtTIFYs* may play a role in abiotic stress and diverse phytohormone-induced responses.

### 2.5. FtTIFYs Expression Patterns under Abiotic Stresses and Hormone Treatments

**Figure 4:**
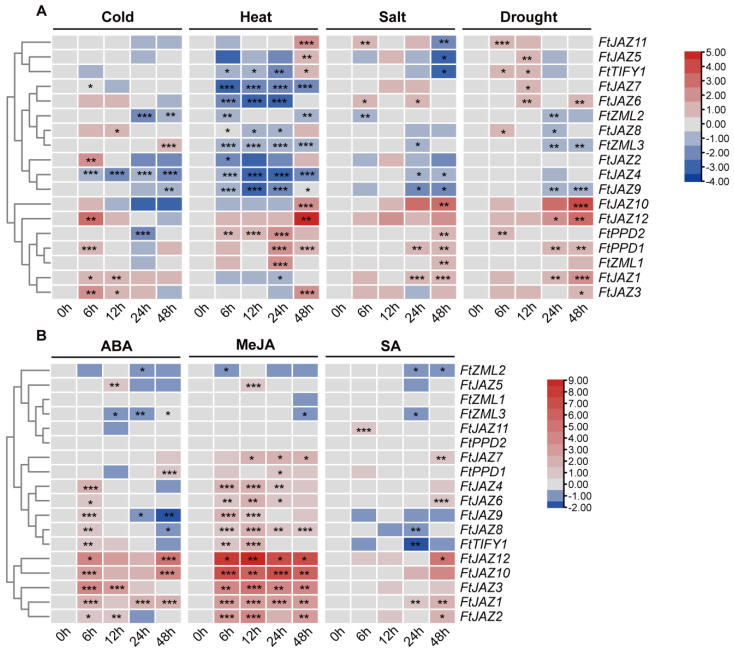
Expression profiles of *FtTIFY* genes under abiotic stresses and hormone treatments. (**A**) Relative expression levels of *FtTIFY* genes under cold (4 °C), heat (37 °C), salt (150 mM NaCl), and drought (30% PEG6000) treatments for 0, 6, 12, 24, and 48 h, respectively. (**B**) Relative expression levels of *FtTIFY* genes under 100 μM abscisic acid (ABA), 2 mM methyl jasmonate (MeJA), and 1 mM salicylic acid (SA) treatments for 0, 6, 12, 24, and 48 h, respectively. Log2-transformed values were used to create the heatmap. The color-based scale bar is shown to the right of each heatmap. Asterisks indicate significant correlations (* *p* < 0.05, ** *p* < 0.01, *** *p* < 0.001; one-way ANOVA). Statistical graphs are shown in Figures S2–S8.

To determine the expression patterns of the *FtTIFY* genes in response to various abiotic stresses or plant hormones, we carried out a qRT-PCR assay on Tatary buckwheat seedlings subjected to different treatments. According to the results, nearly every *FtTIFY* gene showed stress responses to one or more abiotic stimuli, such as cold, heat, salt, and drought. Some of the *FtTIFY* genes exhibited significant expression changes (Figure 4A). Among the genes, three (*FtJAZ2*, *FtJAZ3*, and *FtJAZ12*) were strongly induced by cold, seven (*FtJAZ3*, *FtJAZ10-12*, *FtPPD1*, *FtPPD2*, and *FtZML1*) by heat, two (*FtJAZ10* and *FtJAZ12*) by salt, and three (*FtJAZ1*, *FtJAZ10*, and *FtJAZ12*) by drought, with the expression level being up-regulated over twofold. Notably, at least one-fold up-regulation of *FtJAZ1*, *FtJAZ3*, *FtJAZ10*, *FtJAZ12*, and *FtPPD1* was observed across three to four stress treatments. However, a number of *FtTIFY* genes were significantly suppressed by multiple stress conditions. For example, *FtJAZ4* was repressed by cold, heat, and salt, whereas *FtJAZ9* and *FtZML2* were repressed by all four stress treatments.

For the hormone treatments, all the *FtTIFY* genes except for *FtPPD2* showed significant responses to ABA, MeJA, and SA (Figure 4B). The expression levels of four genes (*FtJAZ1, FtJAZ2, FtJAZ6*, and *FtJAZ12*) were up-regulated by all three hormones, whereas *FtJAZ4, FtJAZ5,* and *FtPPD1* were only slightly induced by ABA and MeJA. Moreover, in contrast to *FtZML2* and *FtZML3,* whose expression was repressed by ABA, MeJA, and SA, *FtZML1* may particularly be inhibited by MeJA. Interestingly, ABA initially stimulated the expression of *FtJAZ4* after 6 h treatment, but 42 h later, it was repressed.

### 2.6. Subcellular Localization of FtJAZ5, FtJAZ8, FtJAZ10, and FtJAZ12

**Figure 5:**
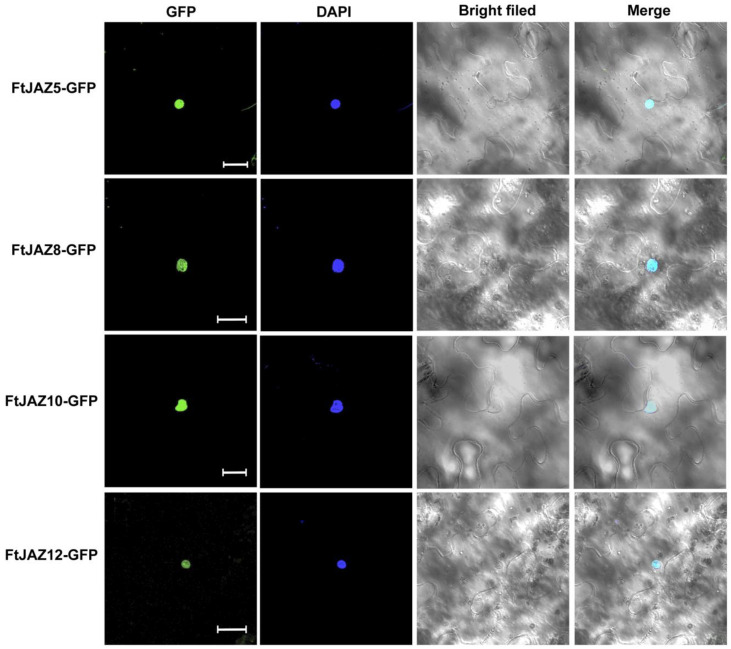
Subcellular localization of FtJAZ5, FtJAZ8, FtJAZ10, and FtJAZ12 in tobacco leaves. Transient expression of FtJAZ5-GFP, FtJAZ8-GFP, FtJAZ10-GFP, and FtJAZ12-GFP fusion proteins in tobacco cells. Bars = 20 μm.

In both *Arabidopsis thaliana* and rice, JAZ proteins were found in the nucleus and played the role of transcriptional repressors [3,18,32]. To determine whether the JAZ homologs in Tartary buckwheat also function in the nucleus, FtJAZ5, FtJAZ8, FtJAZ10, and FtJAZ12 were selected as representative members based on their responses to stresses and phytohormones and were fused to GFP (Green Fluorescence Protein), driven by the CAMV35S promoter. Transient expression in tobacco (*N. tabacum*) leaves showed that the GFP fluorescence signal overlapped with the DAPI-labeled nucleus, indicating that FtJAZs were located in the nucleus (Figure 5).

### 2.7. Interaction Proteins of FtJAZs Assessed via Yeast Two-Hybrid Assays

**Figure 6:**
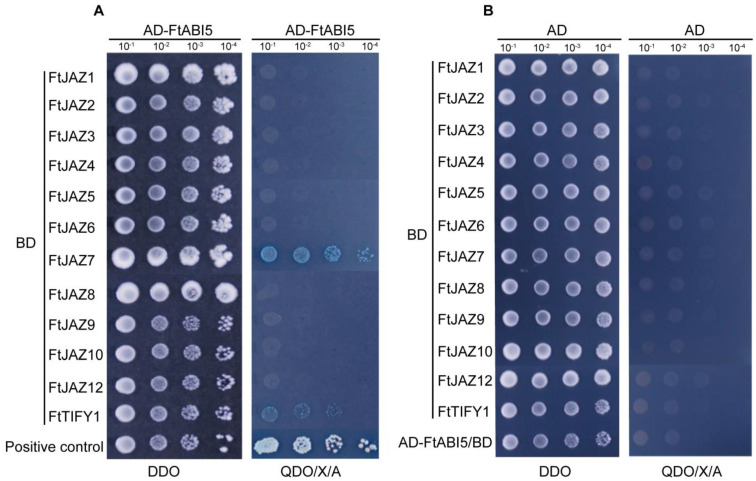
Physical interactions of FtJAZ proteins with FtABI5 assessed via Y2H assay. (**A**) The interaction between FtJAZs (fused with the GAL4-binding domain (BD)) and FtABI5 (fused with the GAL4 activation domain (AD)) was determined via yeast two-hybrid assays. AD-AtDREB2A/BD-AtRCD1 was used as the positive control. (**B**) Empty vector pGADT7 + FtJAZs (fused with the GAL4-binding domain (BD)) and pGBKT7 + FtABI5 (fused with the GAL4 activation domain (AD)) were set as the negative control. The interaction was assessed by yeast growth on DDO (SD/-Trp/-Leu medium) and QDO/X/A (SD/-Trp/-Leu/-His/-Ade medium containing 200 ng/mL of aureobasidin A and 40 μg/mL X-α-Gal). The yeast clones were grown with dilutions to 10^−1^, 10^−2^, 10^−3^, and 10^−4^.

To explore the regulatory mechanism of TIFY proteins in plant stress responses, a protein–protein interaction network (Figure S9) was constructed with the STRING web server [33] based on the previous study of homologous proteins in *Arabidopsis thaliana*. We also noticed that a subgroup of JAZs physically interacted with the ABI5 protein, an important transcription factor in the ABA signaling pathway, and suppressed its transcriptional activity in wheat and *Arabidopsis thaliana* [34]. However, ABI5 was not identified in the interaction network, prompting us to wonder whether FtABI5 interacts with FtJAZs in Tartary buckwheat. To this end, 11 *FtJAZ* genes and their closest homolog, *FtTIFY1*, were cloned and fused to the GAL4 DNA-binding domain as baits, and FtABI5 was ligated to the GAL4 activation domain as prey for yeast two-hybrid assays. The results showed that the FtJAZ7-FtABI5 and FtTIFY1-FtABI5 combinations, as well as the positive controls, were able to grow on the plate of DDO (SD/-Trp/-Leu) and QDO/X/A (SD/-Trp/-Leu/-His/-Ade + X-α-Gal + Aureobasidin A) (Figure 6A), while the other combinations or the negative control group did not grow on the plate of QDO/X/A (Figure 6B), which indicated that both FtJAZ7 and FtTIFY1 could directly interact with FtABI5 in vivo.

### 2.8. Overexpression of FtJAZ10 and FtJAZ12 Increases Heat Tolerance in Tobacco Leaves

**Figure 7:**
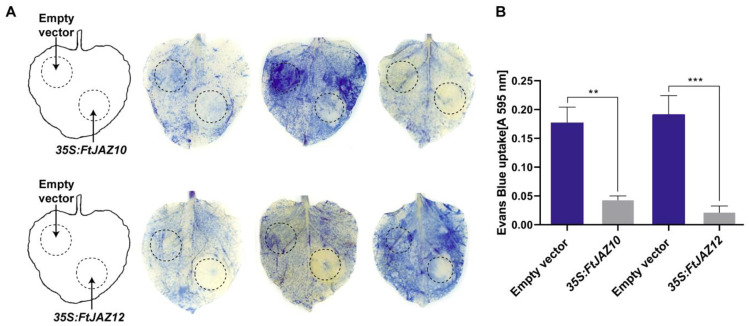
Heat-induced cell death following transient overexpression of *FtJAZs* in tobacco leaves. (**A**) Images of Evans blue staining of tobacco leaves with empty vector and transiently expressed *35S:FtJAZ10/35S:FtJAZ12* under heat stress. (**B**) The extent of cell death was estimated by spectrophotometrically monitoring the retention of Evans blue. The error bars indicate SEM (*n* = 4). One-way ANOVA (Tukey’s test) was performed, and statistically significant differences are indicated by different asterisks (** *p* < 0.01, *** *p* < 0.001).

Since the majority of *FtTIFYs* were activated by various abiotic stresses, we questioned whether the overexpression of *FtTIFY* genes could render plants more resistant to stresses. Hence, *FtJAZ10* and *FtJAZ12* were selected as representative genes for the stress tolerance assay, considering their high expression levels in response to heat stress treatments in the qRT-PCR analysis. They were transiently expressed in tobacco leaves injected with *Agrobacterium* harboring either a *FtJAZ* overexpression or an empty vector. The stress tolerance assay was performed with heat treatment at 48 °C for 1.5 h. The results showed that the Evans blue staining of the area overexpressing *FtJAZ10* or *FtJAZ12* was significantly reduced compared to that of the empty control area in the same leaf (Figure 7), which means that heat-induced cell death was suppressed, suggesting that the transient overexpression of *FtJAZ10* and *FtJAZ12* improved stress tolerance in tobacco.

## 3. Discussion

The *TIFY* gene family was reported to play crucial roles in development and responses to abiotic stresses in many plant species [35], such as *Arabidopsis thaliana* [1], *Oryza sativa* [17], *Vitis vinifera* [8], and *Ficus carica* [36]. However, our understanding of *TIFY* genes in Tartary buckwheat remains limited, leading to incomplete knowledge of the plant’s behavior in response to external stimuli or hormone-signaling transduction. Therefore, in this study, we aimed to identify and analyze all the TIFY-domain-containing genes in Tartary buckwheat, elucidating their sequence characteristics, expression patterns, and potential functions in enhancing plant stress tolerance and transducing phytohormone signaling. All the above-mentioned findings of this research provide valuable insight into the molecular mechanisms of key TIFY family members involved in plant growth and stress tolerance while also identifying potential candidate genes for molecular breeding purposes.

### 3.1. Structural Features and Evolution of FtTIFYs

In Tartary buckwheat, eighteen *TIFY* genes (Table 1), which were evenly distributed in eight chromosomes, were identified. According to the domain composition and phylogenetic studies, the TIFY proteins of Tartary buckwheat were categorized into four subfamilies (TIFY, JAZ, ZML, and PPD). Noteworthy, the FtTIFY1 protein was assigned within the JAZ I group in the phylogenetic tree (Figure 1), suggesting its probable origin as a JAZ protein that subsequently underwent evolutionary modifications, causing the loss of the Jas domain. As a result, it may exhibit similar functions to its closest homolog, FtJAZ7. However, it is important to consider the possibility of misinformation caused by mutations or incompleteness of the genome draft. Therefore, it is necessary to validate the gene’s structure using more accurate genome data in forthcoming studies.

Gene duplication is important for plant functional diversity and evolutionary mechanisms [37]. In this study, only one pair of genes, *FtTIFY1* and *FtJAZ4*, displayed the segmental duplication event in the Tartary buckwheat genome (Figure 2A), indicating that each of the *FtTIFYs* was relatively independent in the evolution process. The synteny analysis of *TIFY* genes showed that the greatest number of gene pairs identified were those between Tartary buckwheat and soybean (Figure 2B). Conversely, only one syntenic gene pair between *FtTIFYs* and *TIFYs* was found in monocotyledonous plants such as rice and maize. These results demonstrate the conservation of *TIFY* genes in dicots and establish a closer evolutionary relationship between *FtTIFYs* and soybean homologs, providing further support to the notion that Tartary buckwheat and soybean share a more direct ancestry [24].

Exon/intron variation among members of a gene family is crucial in the evolution of multigene families [38]. The *FtTIFY* genes contained one to twelve introns, and several sets of *FtTIFYs* in the same clade shared comparable exon/intron structures (Figure 3C), suggesting that they may be the result of duplication events. In particular, *FtJAZ2*, *FtJAZ3*, *FtJAZ10*, and *FtJAZ12* each had a single intron, and given that *JAZs* containing fewer introns in the same subgroup respond more rapidly to stresses [39,40], these four *JAZs* may play crucial roles in stress responses. In addition, exon/intron gain was identified by comparing *FtZML2* to other *FtZML* genes and *FtJAZ11* to the remaining *FtJAZs*. This may have occurred as a consequence of exon splicing, exon duplication, recombination, transposition, or retroposition during the evolution of the *FtTIFY* gene family [30,38].

### 3.2. Expression Profiles of FtTIFYs under Abiotic Stresses and Hormone Treatments

*Cis*-acting elements in promoter sequences serve crucial functions in controlling how plants react to environmental stresses [41]. The prediction of the *FtTIFY* gene promoters revealed the presence of hormone-related *cis*-acting elements that control the responses to ABA, MeJA, and SA (Figure 3D). In addition, a wide variety of stress-responsive elements, such as those that react to low temperatures, drought, wounds, and defense/stress, were also identified. This suggested that *TIFY* genes may be involved in important steps along the stress resistance and hormone signaling pathways of Tartary buckwheat.

Indeed, the response of TIFY proteins to various abiotic stresses and hormones in several plants, such as rice, maize, grape, and *Arabidopsis thaliana*, has been extensively documented [1,8,17,42]. In this study, nearly every *FtTIFY* gene showed stress responses to one or more abiotic stimuli, such as cold, heat, salt, and drought, suggesting that *FtTIFYs* play essential roles in adaptation to environmental stresses. Notably, at least one-fold up-regulation of *FtJAZ1*, *FtJAZ3*, *FtJAZ10-12*, *FtPPD1*, and *FtPPD2* was observed across three to four stress treatments. These genes are potential targets for further research on the ways in which Tartary buckwheat reacts to abiotic stress. *FtJAZ10* and *FtJAZ11* were induced by heat, salt, and drought stresses but inhibited by cold stress, while *FtJAZ1* was up-regulated in response to cold, salt, and drought stresses but down-regulated in response to heat stress, which indicates that *FtTIFYs* have different biochemical and molecular functions under different stresses. Moreover, the expression of *FtJAZ3* and *FtJAZ12* was induced far earlier by cold (in 6 h) than by heat (in 48 h), which suggests that Tartary buckwheat responds to cold and heat through fundamentally different signal pathways. It is interesting to note that *FtJAZ4* and *FtJAZ9*, unlike the other *FtJAZs*, may operate as negative regulators in response to abiotic stresses, as they were repressed by most of the stresses.

JAZ proteins are essential transcriptional inhibitors of the JA signaling pathway, and their degradation activates the transcription of JA-responsive genes in response to jasmonic acid stimulation [43]. In *Arabidopsis thaliana*, the JAZ proteins were mainly involved in the jasmonic acid signal transduction pathway, interacting with transcription factors such as COI1, MYC2, NINJA, MYB, and bHLH. JA treatment also rapidly promotes the expression of *JAZ* genes [43]. In our study, almost all the *FtJAZ* genes were significantly up-regulated by MeJA treatment (Figure 4B), which is consistent with the notion that a negative feedback loop exists to replenish the pool of *JAZs* and attenuate the response to JA [8,9,10,11]. Additionally, nearly all the *FtJAZs* were induced by the ABA treatment, indicating that the FtJAZ proteins engaged in the ABA-dependent signal pathway, most likely via their target protein MYC/MYB/ABI5, as reported in other plant species [14,44,45,46,47]. Given that the majority of these *FtJAZs* also responded to SA treatment, they might contribute to the activation of SA-dependent defense. In contrast to *FtJAZs*, the expression of *FtPPDs* and *FtZMLs* was minimally or not affected by the same treatments. Hence, even though *JAZ*, *PPD*, and *ZML* genes all belong to the same family, their regulation mechanisms appear to be remarkably different.

### 3.3. Potential Function of FtTIFYs under Abiotic Stress Treatment

The expression data from the qRT-PCR analysis inspired our hypothesis regarding the crosstalk between JAZ and plant stress adaptation, particularly in the ABA signaling pathway. With this motivation, we delved deeper into the relationship between JAZ and plant stress adaptation, specifically within the context of the ABA signaling pathway.

However, in our investigation, the TIFY protein interaction network analysis revealed various interactions (Figure S9), including those of JAZ subfamily proteins with COI1 and MYC transcription factors, providing evidence of their involvement in downstream signaling [43,48]. However, conspicuously absent from the network was the interaction between JAZs and core ABA signaling components. Of particular interest is ABI5, a crucial transcription factor in the ABA signaling pathway, renowned for its role in regulating plant stress adaptation. Furthermore, previous studies in *Arabidopsis thaliana* and wheat revealed interactions between ABI5 and several JAZs [34], underscoring the importance of investigating the interactions between JAZs and ABA signaling components.

To address this gap, we conducted a yeast two-hybrid analysis, which led to an important discovery. Specifically, only FtJAZ7 and its closest homolog, FtTIFY1, were found to interact with FtABI5 (Figure 6). This result firmly establishes these two proteins as a connection between the two signaling pathways and suggests their potential role in suppressing FtABI5’s transcriptional activity in Tartary buckwheat.

Meanwhile, this finding prompts an intriguing question: do the remaining FtJAZs possess additional crosstalk mechanisms with the ABA signaling pathway? Further research on the potential crosstalk mechanisms of FtJAZs with the ABA signaling pathway, such as their interactions with MYB/MYC, is an exciting avenue for exploration. The revelation of these mechanisms will enhance our knowledge of how plants adapt and respond to environmental stresses.

When the stress-inducible genes *FtJAZ10* and *FtJAZ12* were overexpressed in tobacco leaves driven by the CaMV35S promoter, the transgenic parts of the leaves showed increased heat tolerance compared with the control parts (Figure 7). In rice, *HTG3* (*heat tolerance gene on chromosome 3*) encodes a heat shock factor that was shown to be heat-inducible and strongly correlated with heat tolerance. A large number of *HTG3*-regulated genes were found to be involved in heat shock and jasmonic acid signaling. *OsJAZ9*, one of the heat-responsive *JAZ* genes directly up-regulated by HTG3a, was found to positively regulate heat tolerance [49]. Thus, it is possible that FtJAZ10 and FtJAZ12 are involved in HTG3-mediated thermotolerance in Tartary buckwheat through jasmonic acid signaling. To better understand the roles that each member of the *TIFY* gene family plays, we need to conduct additional research on the *FtTIFY* genes and subject them to a variety of abiotic stresses.

## 4. Materials and Methods

### 4.1. TIFY Genes’ Identification and Sequence Analysis in Tartary Buckwheat

All TIFY family members of Tartary buckwheat were identified using two different methods. First, the Hidden Markov Model (HMM) profile of the TIFY domain (PF06200) was retrieved from the Pfam database (http://pfam.sanger.ac.uk/, accessed on 16 January 2022) [50]. The database (http://www.mbkbase.org/Pinku1/, accessed on 16 January 2022) of the Tartary buckwheat genome was searched for domains with *E*-value < 1 × 10^−5^ using the HMMER 3.3 software system [24,51]. In addition, 18 *Arabidopsis thaliana* [1] and 20 rice TIFY [17] protein sequences were utilized as queries to search TIFY proteins in the Tartary buckwheat genome with BLASTP (*E*-value < 1 × 10^−5^) [52]. Then, the two candidate sets were intersected, and the conserved domains of the candidate proteins were further identified using the Pfam database. Expasy ProtParam (https://web.expasy.org/protparam/, accessed on 16 January 2022) was used to estimate each anticipated TIFY protein’s molecular weight and pI. The TIFY motifs for each protein were determined using MEME [53].

### 4.2. Phylogenetic Analysis

A total of 56 TIFY proteins from *Arabidopsis thaliana* (18 TIFYs), rice (20 TIFYs), and Tartary buckwheat (18 TIFYs) were multiple sequences aligned with MUSCLE [54]. The phylogenetic tree was created employing the maximum likelihood (ML) method via IQtree [55]. The best-fit model was determined using ModelFinder to be JTT + F + R4 with free rate heterogeneity [56]. A total of 1000 bootstrap samplings were run using Ultrafast Bootstrap approximation. The phylogenetic tree was visualized using iTOL (https://itol.embl.de/, accessed on 10 February 2022) [57].

### 4.3. Chromosomal Distribution, Gene Duplication, and Synteny Analysis

The locations of the *FtTIFY* genes on the chromosomes were determined using the Tartary buckwheat genome database with TBtools software [58]. TBtools was also used to identify the duplication events of the *FtTIFY* genes [58]. The syntenic relationships between the *FtTIFY* genes and the *TIFY* genes from the other seven plants (*Arabidopsis thaliana*, rice, maize, soybean, beet, tomato, and sunflower) were determined using the dual synteny plot (*E*-value < 1 × 10^−5^) in TBtools [58]. The nonsynonymous substitution rate (Ka) and synonymous substitution rate (Ks) for repeated *FtTIFY* genes were calculated using the Simple Ka/Ks Calculator in TBtools [58]. 

### 4.4. Sequence Analysis

The conserved motifs were examined with MEME (https://meme-suite.org/meme/tools/meme, accessed on 12 February 2022) with the maximum number of motifs set as 6 [53]. The 2 kb genomic DNA sequences in front of the ATG codon of the *FtTIFYs* were provided to analyze the *cis*-acting elements using PlantCARE (http://bioinformatics.psb.ugent.be/webtools/plantcare/html/, accessed on 12 February 2022) [31]. All the patterns of conserved motifs, exon/intron structures, and the *cis*-acting elements around the promoters were displayed using TBtools software [58].

### 4.5. Plant Material and Treatments

Our study investigated the cultivar “GUIMI” of Tartary buckwheat. The seeds were soaked for 30 min at 37 °C in sterile water, and then the soaked seeds were placed on Petri dishes with moist filter paper. The seeds were germinated for two days at 30 °C in the dark. All the seedlings were planted in soil and developed in the greenhouse (21–23 °C, 16-hour light/8-hour dark cycle). Various treatments were performed on 14-day-old seedlings. Cold and heat treatments were carried out by shifting the seedlings to a growth-dedicated chamber at 4 °C or 37 °C. For the salt, drought, abscisic acid (ABA), methyl jasmonate (MeJA), and salicylic acid (SA) treatments, the seedlings were stressed with 150 mM NaCl, 30% PEG (poly (ethylene glycol)) 6000, 100 μM ABA, 2 mM MeJA, and 1 mM SA. The seedlings were treated for 6, 12, 24, and 48 h (h), and untreated seedlings (0 h) served as the control. The leaf and stem samples were collected and preserved at −80 °C.

### 4.6. RNA Extraction and Quantitative Real-Time PCR (qRT-PCR)

Total RNA was processed for extraction using the RNAprep Pure Plant Kit (TIANGEN, Beijing, China), followed by single-stranded cDNA synthesis with an Evo M-MLV RT Premix for qPCR Kit (AGbio, Hunan, China). All processes were followed in accordance with the manufacturer’s guidelines. Primer Premier 5 software was used to design gene-specific qRT-PCR primers. The specified primers are shown in Supplementary Table S1. qRT-PCR was executed with SYBR Green Premix (AGbio) following the manufacturer’s guidelines, and the reactions were performed with an ABI Q5 PCR Real-Time Thermal Cycler (Thermo Fisher Scientific, Wilmington, Massachusetts, USA). The *H3* gene from Tartary buckwheat was utilized as an internal control [59]. The expression level of the genes was calculated using Pfaffl’s method [60].

### 4.7. Subcellular Localization

The CDS sequence of *FtTIFY* genes was amplified and inserted into the pDONR/zeo vector (Thermo Fisher Scientific, Wilmington, Massachusetts, USA) via the BP reaction. The vector-specific primers are provided in Supplementary Table S2. The validated cDNA fragments were then inserted into pMDC83 via LR reaction and connected to the GFP (green fluorescence protein) N-terminal regulated by the CaMV35S promoter. The combination proteins were momentarily expressed in tobacco (*N. tabacum*) leaves via *Agrobacterium*-mediated transformation [61]. Fusion protein expression was monitored via confocal microscopy (Zeiss LSM880, Oberkochen, Germany) after 36 h of hatching in the dark. The nucleus was colored with DAPI.

### 4.8. Protein Interaction Network Analysis

The STRING web server (https://cn.string-db.org/, accessed on 27 May 2023) was used to predict the FtTIFY protein interaction network based on the corresponding homologous proteins in *Arabidopsis thaliana* [33]. The protein interaction network was visualized in Cytoscape software [33].

### 4.9. Yeast Two-Hybrid Assays

The full-length CDS sequences of *FtABI5* and the *FtTIFYs* were amplified and inserted into pGADT7 and pGBKT7 (Takara, Beijing, China), respectively. The vector-specific primers are presented in Supplementary Table S2. The plasmids were multiplied on DDO (SD/-Trp/-Leu medium) after being transformed into yeast strain Y2HGold, and then the yeast cells were examined on QDO/X/A (SD/-Trp/-Leu/-His/-Ade medium containing 200 ng/mL of aureobasidin A and 40 μg/mL X-α-Gal). Yeast transformation and screening were based on the manufacturer’s guidelines.

### 4.10. Stress Treatment and Evans Blue Leaves Assay

The empty vector and the fusion expression vector pMDC83-*FtJAZ10*/pMDC83-*FtJAZ12* were transiently communicated in tobacco leaves via *Agrobacterium*-mediated transformation [61]. After 60 h of incubation in the dark, the isolated leaves were exposed to heat treatment at 48 °C for 1.5 h. After the stress treatment, the leaves were completely stained with 0.25% (*w*/*v*) Evans blue for 20 min followed by washing with water. To remove the chlorophyll, stained leaves were placed in 95% ethanol for 10 min, and then the leaves were photographed [62]. For the quantitative assessments, taking each stained leaf, we cut out a 9 mm leaf disc from the transformation area, and the Evans blue dye was extracted at 50 °C with 1% (*w*/*v*) SDS in 50% (*v*/*v*) methanol for 1 h. Then, the absorbance of the samples was measured at 595 nm [63].

## 5. Conclusions

In this study, 18 *TIFY* genes were identified in the Tartary buckwheat genome. All of them had TIFY conserved domains, and 17 FtTIFYs, except FtTIFY1, had a Jas conserved motif. Synteny analysis showed that only one pair of genes in the *FtTIFY* genome displayed the segmental duplication event, demonstrating the high conservation of the *TIFY* family. Additionally, Tartary buckwheat is more closely related to dicots, especially soybeans. The classification of the *FtTIFY* genes into eight groups was supported by their conserved domains. Promoter analysis revealed that the majority of the *FtTIFYs* contained hormone- and stress-responsive elements. The expression profiles of the *FtTIFYs* under four abiotic stresses and phytohormone treatments confirmed that *FtTIFYs* play critical roles not only in hormone signaling transduction but also in the response to abiotic stress. Furthermore, two TIFY proteins, FtJAZ7 and FtTIFY1, interacted with FtABI5 in the yeast two-hybrid analysis, indicating the existence of crosstalk between ABA and JA signaling. Moreover, the overexpression of the two most stress-inducible *FtTIFYs*, *FtJAZ10* and *FtJAZ12,* improved the heat tolerance of tobacco, implying the potential significance of *FtTIFYs* for molecular breeding. The findings of this study provide a bioinformatic and molecular foundation for future research on the roles of *FtTIFYs* and candidate genes in the cultivation of stress-resistant crops.

## Figures and Tables

**Table 1:** Basic information of the *TIFY* gene family in Tartary buckwheat.

Gene Name	Gene ID	Genomic Length (bp)	CDS Length (bp)	Protein Length (aa)	MW (kDa)	pI	TIFY Motif
*FtTIFY1*	FtPinG0008957700.01	2368	498	165	17.91	7.67	TIFYGG
*FtPPD1*	FtPinG0001466800.01	4288	903	300	33.33	8.62	TIFYCG
*FtPPD2*	FtPinG0007574300.01	5109	1059	352	38.24	8.60	TIFYCG
*FtZML1*	FtPinG0005620000.01	3071	909	302	32.93	6.52	KILYNV
*FtZML2*	FtPinG0005619800.01	3924	882	293	32.06	6.30	KIRYSV
*FtZML3*	FtPinG0002885200.01	4005	888	295	32.44	6.28	KIRYTV
*FtJAZ1*	FtPinG0008023100.01	1874	693	230	25.10	8.31	TIFYGG
*FtJAZ2*	FtPinG0000834700.01	585	369	122	13.68	8.64	TIFYNG
*FtJAZ3*	FtPinG0001783400.01	636	465	154	17.08	11.00	TIFYNG
*FtJAZ4*	FtPinG0006092000.01	2170	636	211	23.27	9.46	TIFYAG
*FtJAZ5*	FtPinG0001151300.01	2575	813	270	29.53	9.42	TIFYGG
*FtJAZ6*	FtPinG0005124100.01	2374	750	249	27.11	9.55	TIFYAG
*FtJAZ7*	FtPinG0005522500.01	2671	813	270	29.20	10.78	TIFYAG
*FtJAZ8*	FtPinG0000682400.01	2588	669	222	24.34	9.75	TIFYGG
*FtJAZ9*	FtPinG0000682800.01	1534	678	225	24.46	10.00	TIFYGG
*FtJAZ10*	FtPinG0006744800.01	455	345	114	12.91	9.63	TIFYNG
*FtJAZ11*	FtPinG0003387600.01	2974	1665	554	57.47	10.26	QIRFSQ
*FtJAZ12*	FtPinG0001004100.01	638	381	126	14.25	10.02	TIIYNG

## Data Availability

The data presented in this study are available in this article and its Supplementary Materials.

## Supplementary Materials

The following supporting information can be downloaded at: https://www.mdpi.com/article/10.3390/ijms241310916/s1.
