# Supplementary material for: Genome-Wide Identification and Functional Analysis of the TIFY Family Genes in Response to Abiotic Stresses and Hormone Treatments in Tartary Buckwheat (Fagopyrum tataricum)
Source: Int J Mol Sci. 2023 Jun 30;24(13):10916. doi: 10.3390/ijms241310916 (PMC10341436; doi:10.3390/ijms241310916)
Supplement: Supplementary file 1 [file ijms-24-10916-s001.zip › Figures S1–S9.pdf]

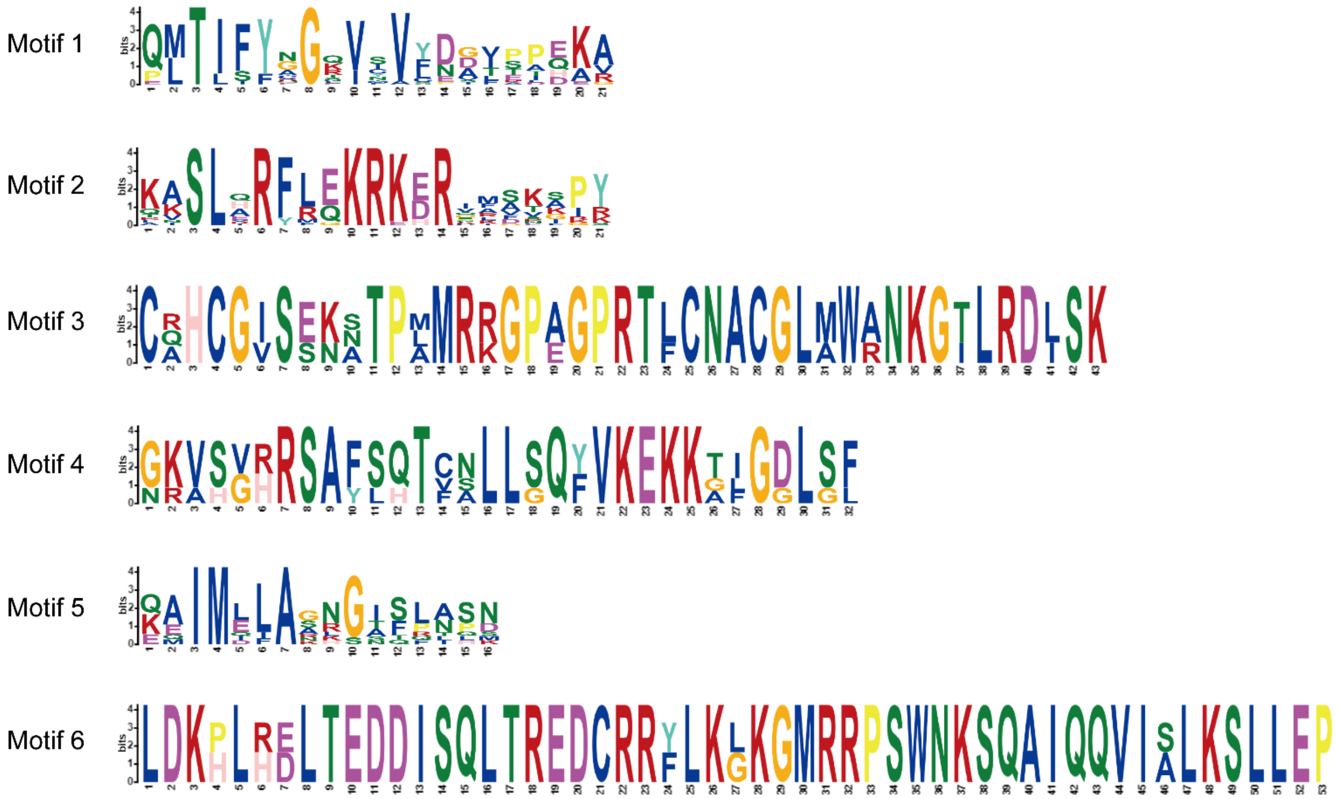

Figure S1. Protein motifs identified in 18 FtTIFY proteins through MEME.

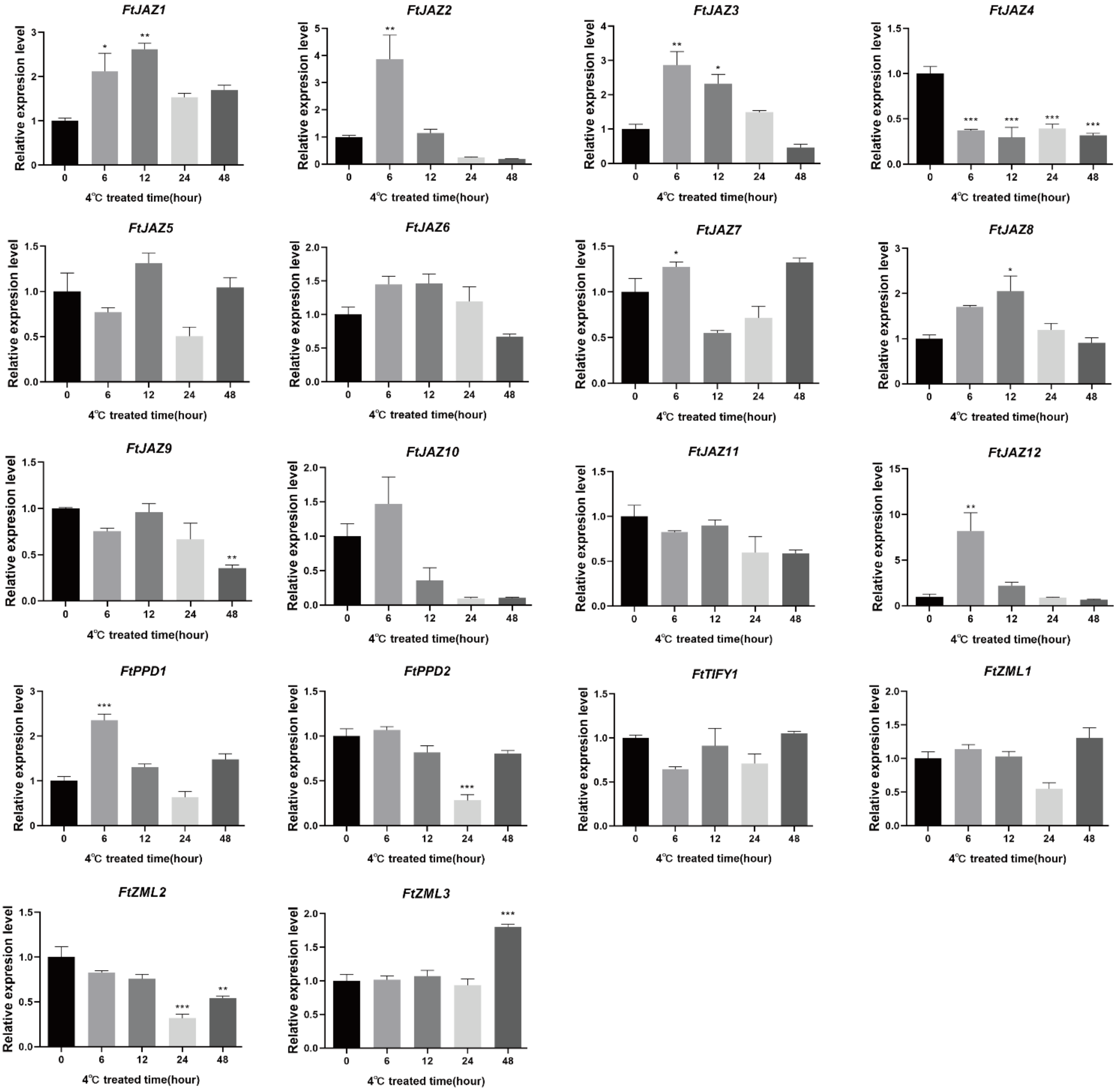

Figure S2. Expression profiles of *FtTIFY* genes under cold stress. The expression level at 0 h was defined as 1.0, and the expression levels at other time points were normalized accordingly. *FtH3* was used as the internal control for qRT-PCR analysis. Data were presented as the average of the three technical replicates, error bars represent SEM (n = 3). One-way ANOVA (Tukey's test) was performed, and statistically significant differences are indicated by different asterisks (* $p < 0.05$, ** $p < 0.01$, *** $p < 0.001$).

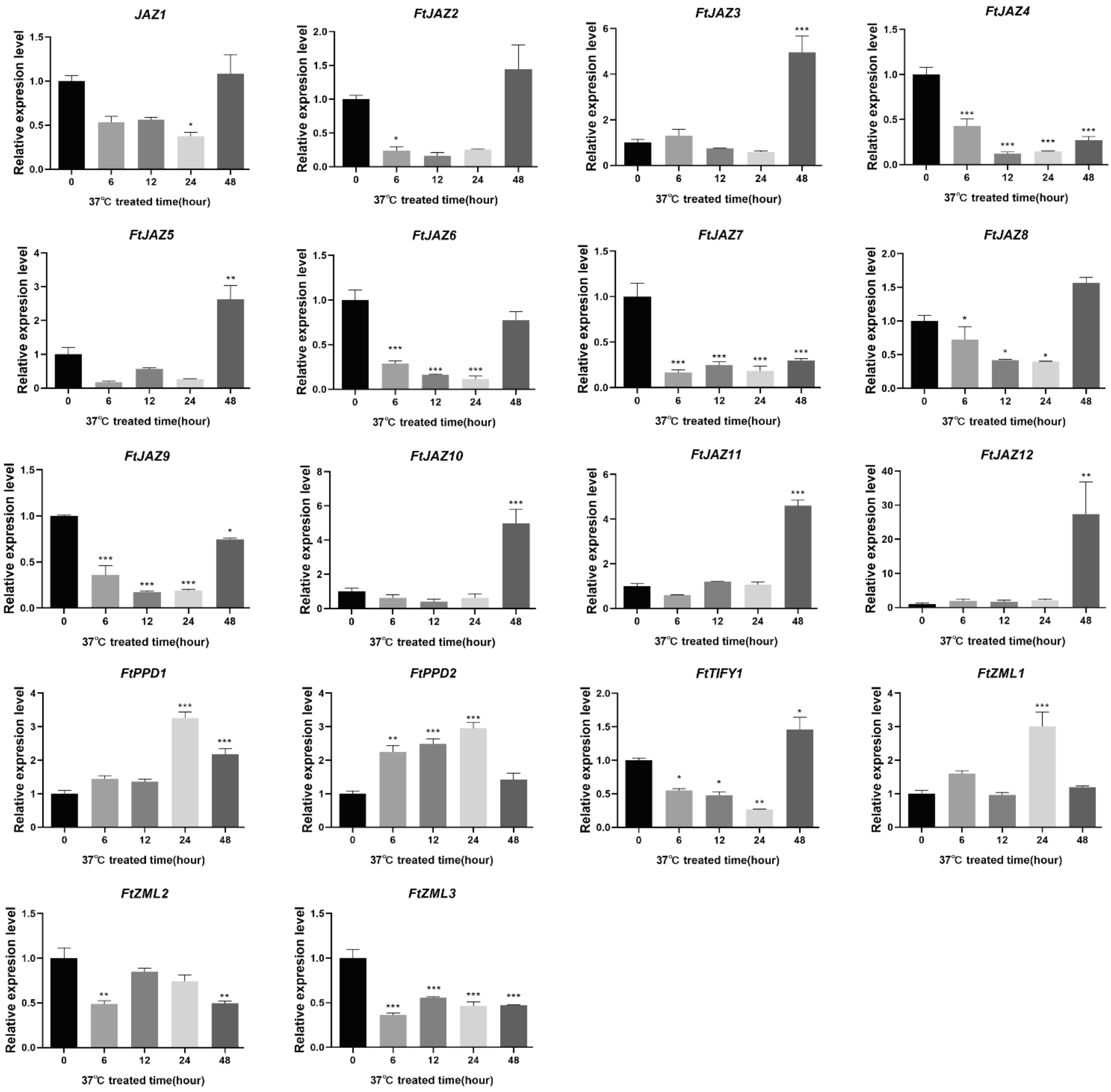

Figure S3. Expression profiles of *FtTIFY* genes under heat stress. The expression level at 0 h was defined as 1.0, and the expression levels at other time points were normalized accordingly. *FtH3* was used as the internal control for qRT-PCR analysis. Data were presented as the average of the three technical replicates, error bars represent SEM (n = 3). One-way ANOVA (Tukey's test) was performed, and statistically significant differences are indicated by different asterisks (* $p < 0.05$, ** $p < 0.01$, *** $p < 0.001$).

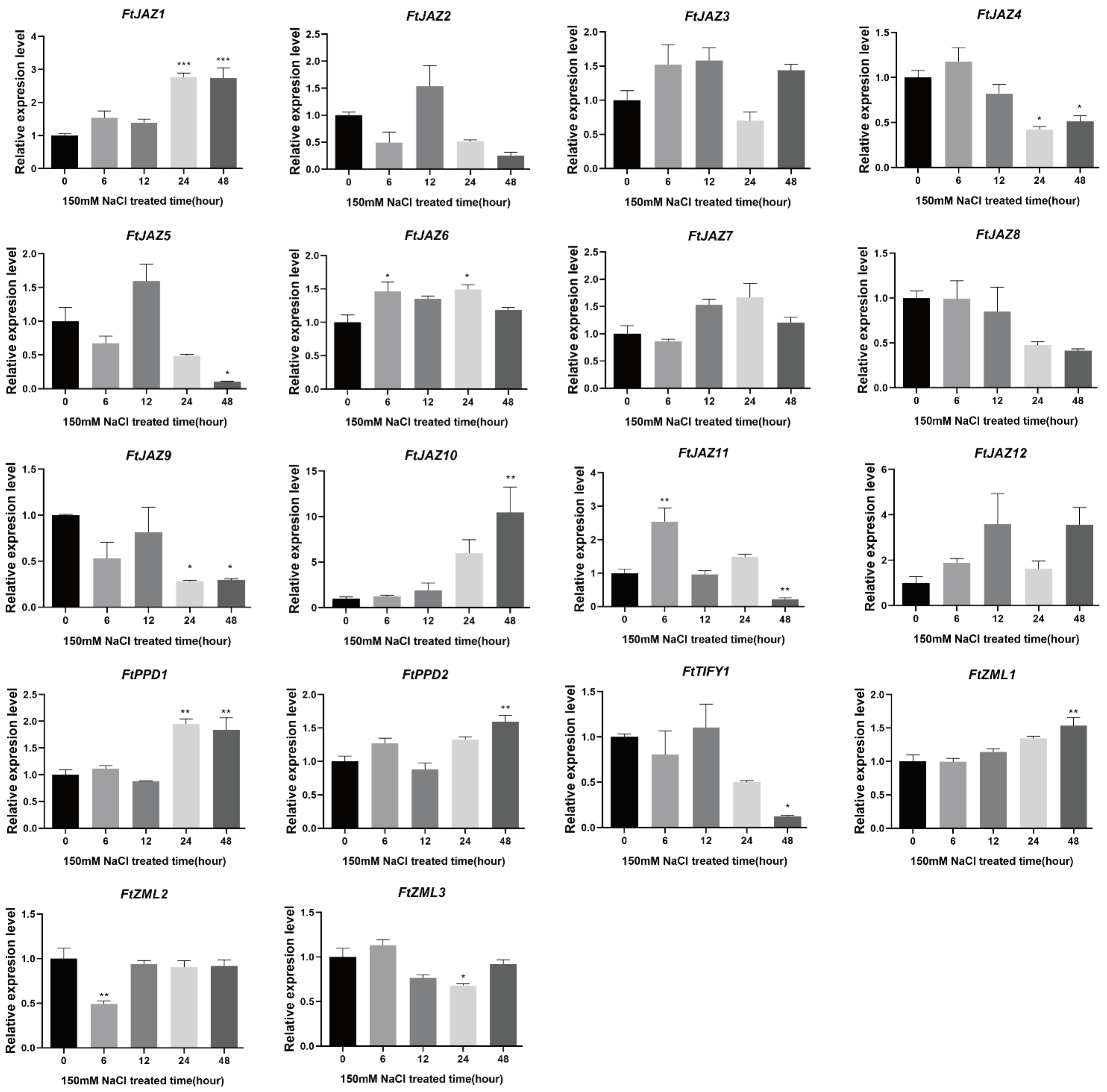

Figure S4. Expression profiles of *FtTIFY* genes under salt stress. The expression level at 0 h was defined as 1.0, and the expression levels at other time points were normalized accordingly. *FtH3* was used as the internal control for qRT-PCR analysis. Data were presented as the average of the three technical replicates, error bars represent SEM (n = 3). One-way ANOVA (Tukey's test) was performed, and statistically significant differences are indicated by different asterisks (* $p < 0.05$, ** $p < 0.01$, *** $p < 0.001$).

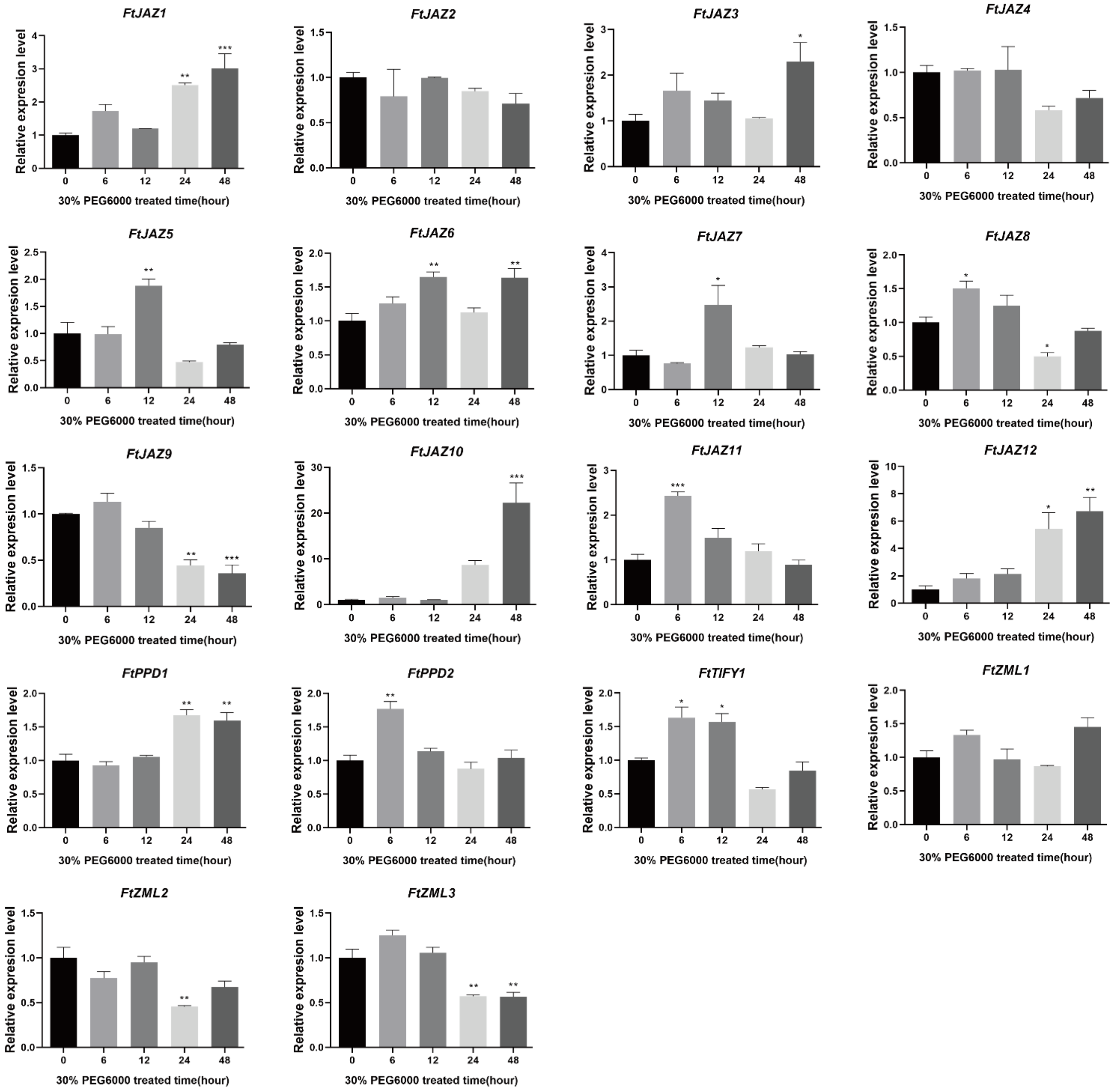

Figure S5. Expression profiles of *FtTIFY* genes under drought stress. The expression level at 0 h was defined as 1.0, and the expression levels at other time points were normalized accordingly. *FtH3* was used as the internal control for qRT-PCR analysis. Data were presented as the average of the three technical replicates, error bars represent SEM (n = 3). One-way ANOVA (Tukey's test) was performed, and statistically significant differences are indicated by different asterisks (* $p < 0.05$, ** $p < 0.01$, *** $p < 0.001$).

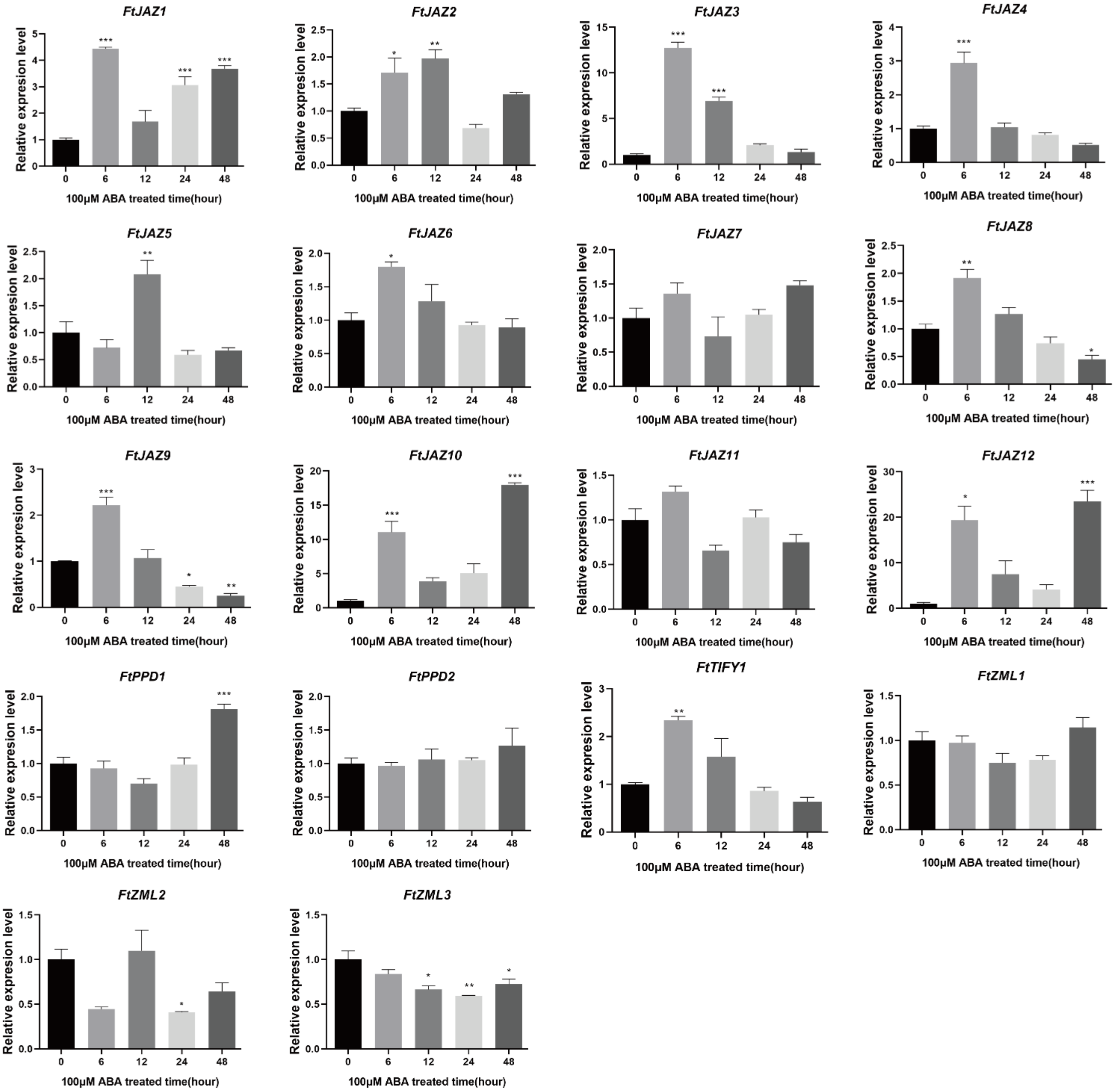

Figure S6. Expression profiles of *FtTIFY* genes under ABA treatment. The expression level at 0 h was defined as 1.0, and the expression levels at other time points were normalized accordingly. *FtH3* was used as the internal control for qRT-PCR analysis. Data were presented as the average of the three technical replicates, error bars represent SEM ($n = 3$). One-way ANOVA (Tukey's test) was performed, and statistically significant differences are indicated by different asterisks (* $p < 0.05$, ** $p < 0.01$, *** $p < 0.001$).

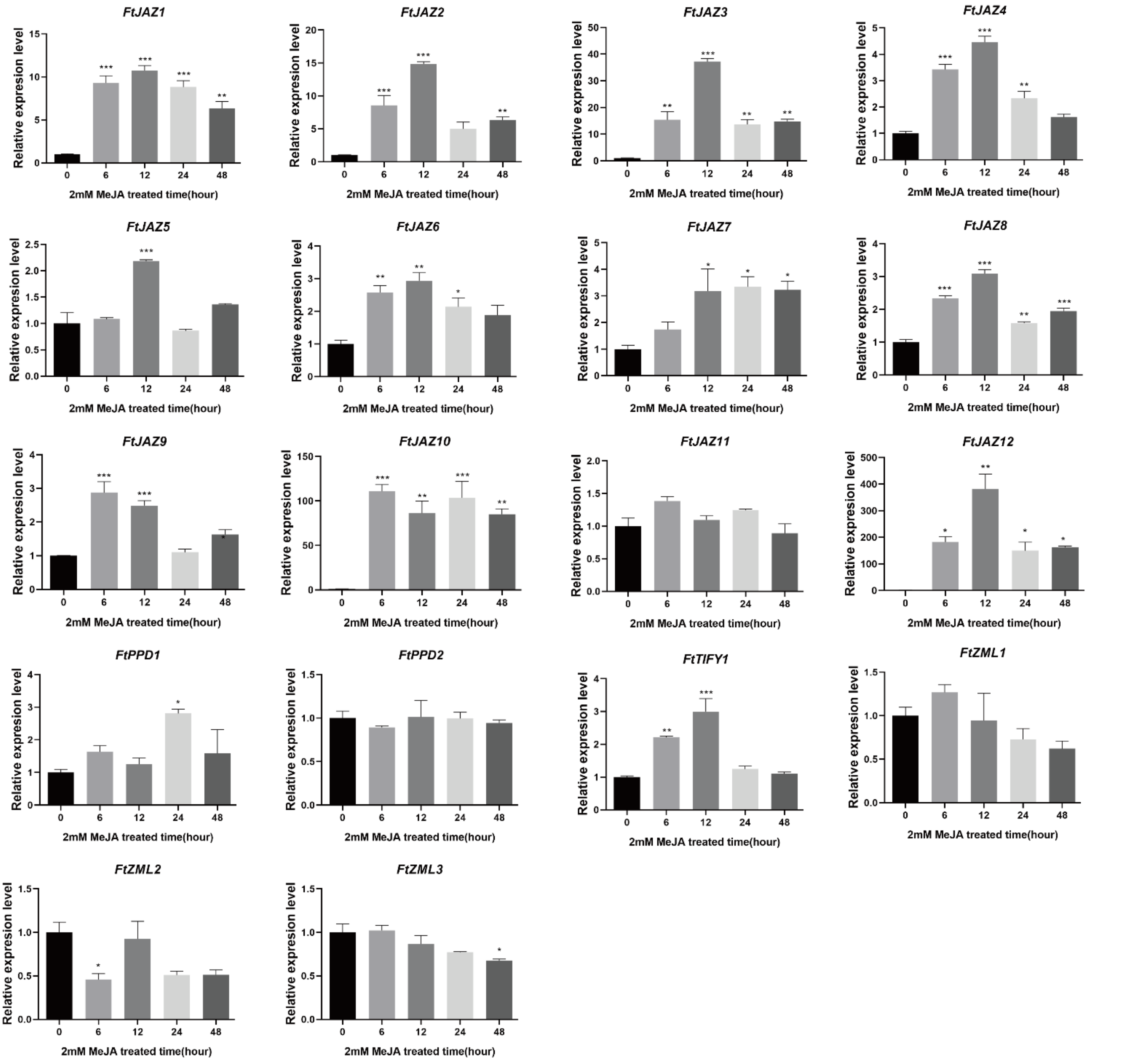

Figure S7. Expression profiles of *FtTIFY* genes under MeJA treatment. The expression level at 0 h was defined as 1.0, and the expression levels at other time points were normalized accordingly. *FtH3* was used as the internal control for qRT-PCR analysis. Data were presented as the average of the three technical replicates, error bars represent SEM ($n = 3$). One-way ANOVA (Tukey's test) was performed, and statistically significant differences are indicated by different asterisks (* $p < 0.05$, ** $p < 0.01$, *** $p < 0.001$).

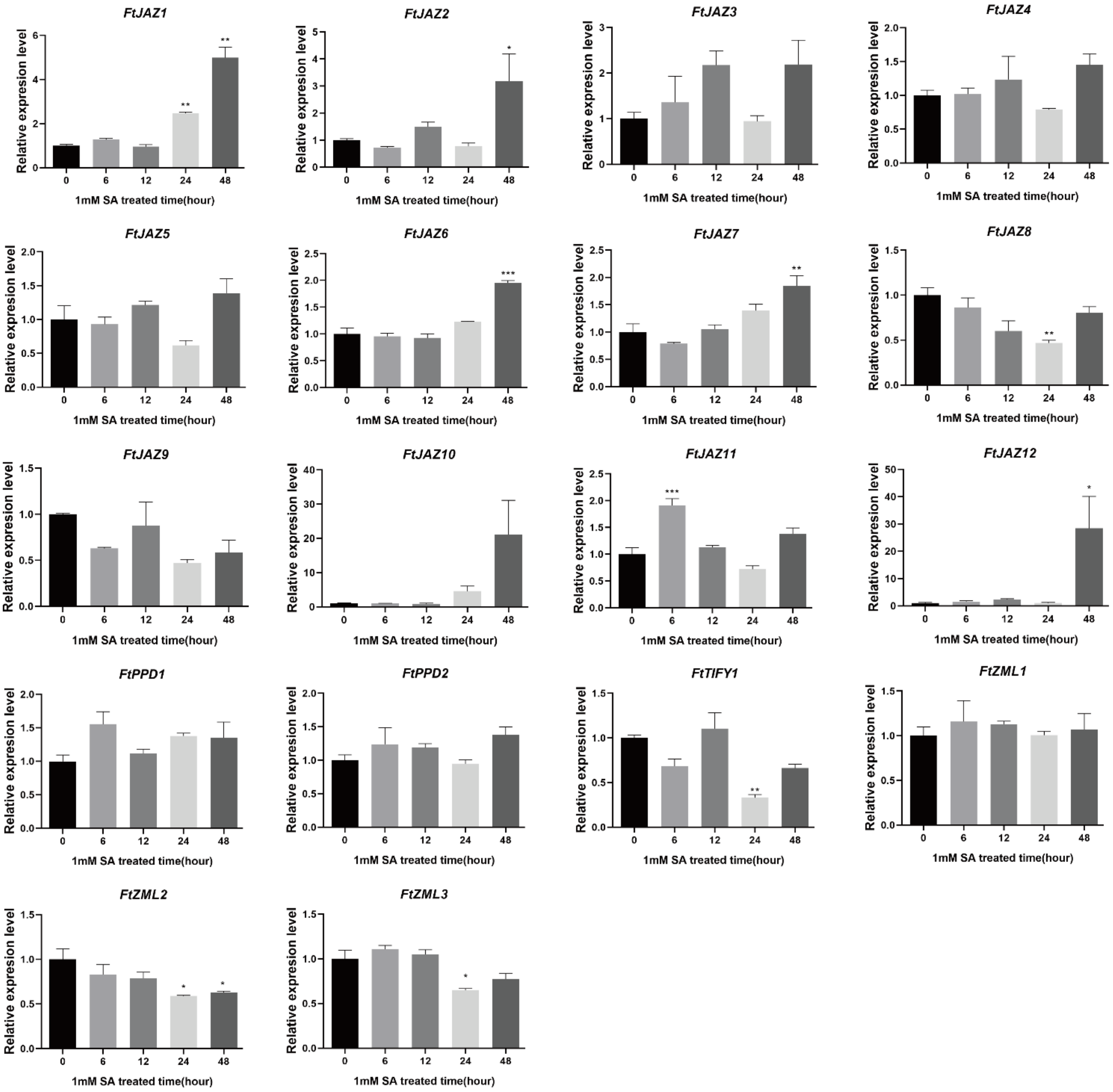

Figure S8. Expression profiles of *FtTIFY* genes under SA treatment. The expression level at 0 h was defined as 1.0, and the expression levels at other time points were normalized accordingly. *FtH3* was used as the internal control for qRT-PCR analysis. Data were presented as the average of the three technical replicates, error bars represent SEM (n = 3). One-way ANOVA (Tukey's test) was performed, and statistically significant differences are indicated by different asterisks (* $p < 0.05$, ** $p < 0.01$, *** $p < 0.001$).

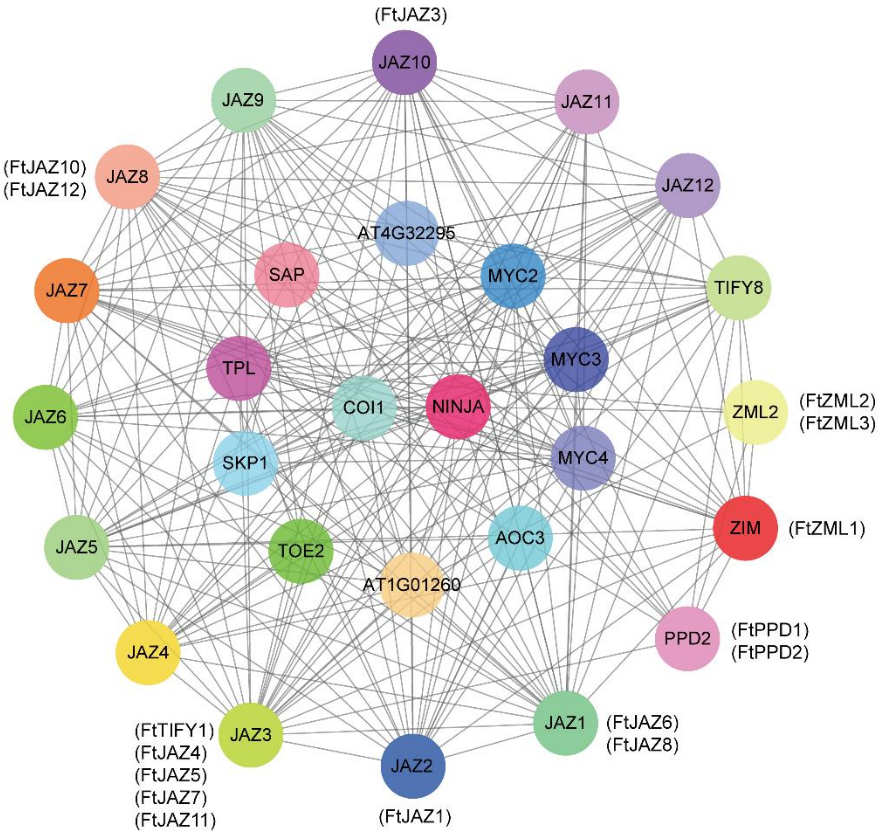

Figure S9. Protein-protein interaction network of FtTIFY proteins according to the orthologs in *Arabidopsis thaliana.*
